# (*E*)-2-(2,3-Dimethyl­anilino)-*N*′-[2-methyl-5-(prop-1-en-2-yl)cyclo­hex-2-enyl­idene]benzohydrazide

**DOI:** 10.1107/S1600536812009087

**Published:** 2012-03-21

**Authors:** Mashooq A. Bhat, Hatem A. Abdel-Aziz, Hazem A. Ghabbour, Madhukar Hemamalini, Hoong-Kun Fun

**Affiliations:** a Department of Pharmaceutical Chemistry, College of Pharmacy, King Saud University, PO Box 2457, Riyadh 11451, Saudi Arabia; b X-ray Crystallography Unit, School of Physics, Universiti Sains Malaysia, 11800 USM, Penang, Malaysia

## Abstract

The asymmetric unit of the title compound, C_25_H_29_N_3_O, comprises two crystallographically independent mol­ecules. The dihedral angles between the benzene rings in the two mol­ecules are 59.7 (2) and 61.27 (18)°. The cyclo­hexene rings adopt sofa and half-chair conformations. In the crystal, mol­ecules are connected *via* N—H⋯O and weak C—H⋯O hydrogen bonds, forming chains along the *a* axis. In each mol­ecule, there is an intra­molecular N—H⋯O hydrogen bond.

## Related literature

For background to the chemistry and biological activity of diaryl amines, see: Reddy *et al.* (2010 ▶); Ohta *et al.* (2008) ▶; Li *et al.* (2008) ▶. For related structures, see: Wang *et al.* (2010 ▶); Tian *et al.* (2010 ▶). For ring conformations, see: Cremer & Pople (1975 ▶). For standard bond-length data, see: Allen *et al.* (1987 ▶).
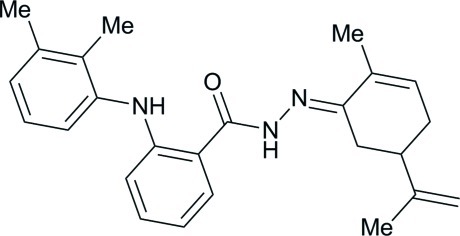


## Experimental

### 

#### Crystal data

C_25_H_29_N_3_O
*M* *_r_* = 387.51
Orthorhombic, 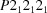

*a* = 9.0296 (4) Å
*b* = 18.0457 (7) Å
*c* = 27.4755 (10) Å
*V* = 4477.0 (3) Å^3^
*Z* = 8
Cu *K*α radiation
μ = 0.55 mm^−1^
*T* = 296 K
10.28 × 0.28 × 0.09 mm

#### Data collection

Bruker SMART APEXII CCD area-detector diffractometer
Absorption correction: multi-scan (*SADABS*; Bruker, 2009 ▶) *T*_min_ = 0.070, *T*_max_ = 0.955
17464 measured reflections
7599 independent reflections
5248 reflections with *I* > 2σ(*I*)
*R*_int_ = 0.027

#### Refinement

*R*[*F* ^2^ > 2σ(*F* ^2^)] = 0.059
*wR*(*F* ^2^) = 0.167
*S* = 1.03
7599 reflections
531 parameters
H-atom parameters constrained
Δρ_max_ = 0.13 e Å^−3^
Δρ_min_ = −0.11 e Å^−3^

### 

Data collection: *APEX2* (Bruker, 2009 ▶); cell refinement: *SAINT* (Bruker, 2009 ▶); data reduction: *SAINT*; program(s) used to solve structure: *SHELXTL* (Sheldrick, 2008 ▶); program(s) used to refine structure: *SHELXTL*; molecular graphics: *SHELXTL*; software used to prepare material for publication: *SHELXTL* and *PLATON* (Spek, 2009 ▶).

## Supplementary Material

Crystal structure: contains datablock(s) global, I. DOI: 10.1107/S1600536812009087/lh5424sup1.cif

Structure factors: contains datablock(s) I. DOI: 10.1107/S1600536812009087/lh5424Isup2.hkl

Supplementary material file. DOI: 10.1107/S1600536812009087/lh5424Isup3.cml

Additional supplementary materials:  crystallographic information; 3D view; checkCIF report

## Figures and Tables

**Table 1:** Hydrogen-bond geometry (Å, °)

*D*—H⋯*A*	*D*—H	H⋯*A*	*D*⋯*A*	*D*—H⋯*A*
N2*A*—H2*AB*⋯O1*B*	0.86	2.31	2.969 (4)	134
N3*A*—H3*AC*⋯O1*A*	0.86	2.14	2.697 (3)	122
N2*B*—H2*BB*⋯O1*A*^i^	0.86	2.36	2.986 (4)	130
C5*A*—H5*AB*⋯O1*B*	0.97	2.47	3.189 (4)	130
C9*A*—H9*AA*⋯O1*B*	0.93	2.43	3.282 (4)	152
C5*B*—H5*BA*⋯O1*A*^i^	0.97	2.46	3.248 (4)	138
C9*B*—H9*BA*⋯O1*A*^i^	0.93	2.50	3.352 (4)	153

Symmetry code: (i) 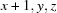.

**Fig. 1.**
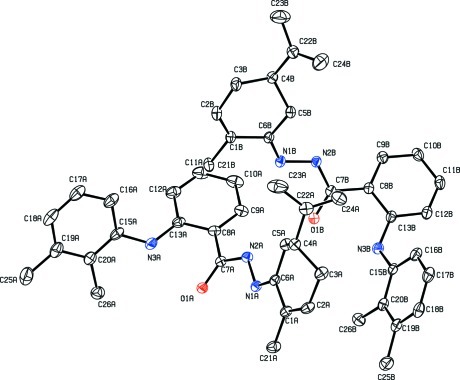
The asymmetric unit of the title compound, showing 10% probability displacement ellipsoids. Hydrogen atoms omitted for clarity.

**Fig. 2.**
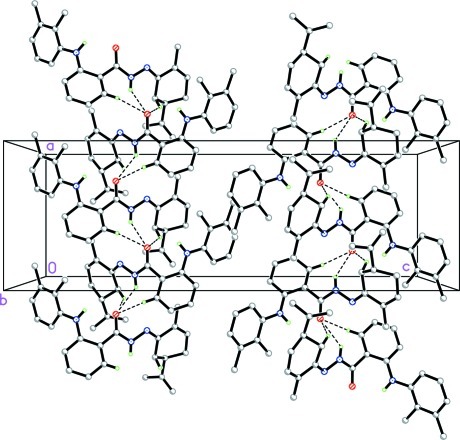
The crystal packing of the title compound (I).

## supplementary crystallographic information

### Comment

Diarylamines represent an important class of compounds due to their wide applications and special pharmacological activities (Ohta *et al.*, 2008; Li *et al.*, 2008; Reddy *et al.*, 2010). The crystal structues of 4-(*o*-Tolylamino) benzaldehyde (Wang *et al.*, 2010) and 4-(*p*-Tolylamino) benzaldehyde (Tian *et al.*, 2010) have been reported in the literature. Herein, we reported the crystal structure of the title compound, (I).

The asymmetric unit of the title compound consists of two crystallographically independent (*E*)-2-(2,3-dimethylphenylamino)- N'-(2-methyl-5-(prop-1-en-2-yl)cyclohex-2-enylidene)benzohydrazide (A & B), as shown in Fig. 1. The bond lengths and angles of molecules A and B agree with each other and are within normal ranges for bond lengths (Allen *et al.*, 1987). The dihedral angles between benzene rings (C8A–C13A)/(C15A–C20A), and (C8B–C13B)/(C15B–C20B) are 59.7 (2)° and 61.27 (18)° ° respectively. In molecule A (C1A–C6A), the cylcohexene rings adopts a sofa conformation [Q = 0.499 (4) Å, θ = 57.1 (5)°, φ = 176.4 (5)°]. In molecule B (C1B–C6B), the cyclohexene ring adopts a half-chair conformation [Q = 0.431 (4) Å, θ = 53.8 (5)°, φ = 195.3 (7)°; Cremer & Pople, 1975].

In the crystal structure (Fig. 2), molecules are connected *via* intermolecular N—H···O and C—H···O hydrogen bonds, forming one-dimensional chains along the *a*-axis. In each molecule the is an intramolecular N—H···O hydrogen bond.

### Experimental

The title compound was prepared by the reaction of carvone, 2-methyl-5-(prop-1-en-2-yl)cyclohex-2-enone (0.15 g, 1 mmol) and 2-(2,3-dimethylphenylamino)benzohydrazide (0.26 g, 1 mmol) in ETOH (25 mL). After stirring for 3 h, at room temperature, the resulting mixture was concentrated. The precipitate washed with ETOH to afford the title compound. Colorless blocks of the title compound suitable for X-ray structure determination were recrystallized from ETOH by the slow evaporation of the solvent at room temperature. The crystals are very brittle and shatter easily upon cutting so data were collected using a crystal which was a long needle.

### Refinement

All H atoms were positioned geometrically [C—H = 0.93–0.98 Å and N—H = 0.86 Å] and were refined using a riding model, with *U*_iso_(H) = 1.2 or 1.5 *U*_eq_(C). A rotating group model was applied to the methyl groups. The standard uncertainty of the refined Flack parameter indicates the result is inconclusive. The structure contains psuedosymmetry but examination of the systematic absences and the inability to refine the structure in the higher symmetry space group confirm the current choice of space group.

### Crystal data

**Table:**

C_25_H_29_N_3_O	*F*(000) = 1664
*M**_r_* = 387.51	*D*_x_ = 1.150 Mg m^−^^3^
Orthorhombic, *P*2_1_2_1_2_1_	Cu *K*α radiation, λ = 1.54178 Å
Hall symbol: P 2ac 2ab	Cell parameters from 6172 reflections
*a* = 9.0296 (4) Å	θ = 2.5–65.3°
*b* = 18.0457 (7) Å	µ = 0.55 mm^−^^1^
*c* = 27.4755 (10) Å	*T* = 296 K
*V* = 4477.0 (3) Å^3^	Plate, colourless
*Z* = 8	10.28 × 0.28 × 0.09 mm

### Data collection

**Table:**

Bruker SMART APEXII CCD area-detector diffractometer	7599 independent reflections
Radiation source: fine-focus sealed tube	5248 reflections with *I* > 2σ(*I*)
Graphite monochromator	*R*_int_ = 0.027
φ and ω scans	θ_max_ = 69.7°, θ_min_ = 2.9°
Absorption correction: multi-scan (*SADABS*; Bruker, 2009)	*h* = −8→10
*T*_min_ = 0.070, *T*_max_ = 0.955	*k* = −12→21
17464 measured reflections	*l* = −33→27

### Refinement

**Table:**

Refinement on *F*^2^	Secondary atom site location: difference Fourier map
Least-squares matrix: full	Hydrogen site location: inferred from neighbouring sites
*R*[*F*^2^ > 2σ(*F*^2^)] = 0.059	H-atom parameters constrained
*wR*(*F*^2^) = 0.167	*w* = 1/[σ^2^(*F*_o_^2^) + (0.0785*P*)^2^ + 0.183*P*] where *P* = (*F*_o_^2^ + 2*F*_c_^2^)/3
*S* = 1.03	(Δ/σ)_max_ < 0.001
7599 reflections	Δρ_max_ = 0.13 e Å^−^^3^
531 parameters	Δρ_min_ = −0.11 e Å^−^^3^
0 restraints	Absolute structure: Flack, H. D. (1983). *Acta Cryst.* A39, 876–881.
Primary atom site location: structure-invariant direct methods	Flack parameter: 0.4 (4)

### Special details

**Table:**

Geometry. All s.u.'s (except the s.u. in the dihedral angle between two l.s. planes) are estimated using the full covariance matrix. The cell s.u.'s are taken into account individually in the estimation of s.u.'s in distances, angles and torsion angles; correlations between s.u.'s in cell parameters are only used when they are defined by crystal symmetry. An approximate (isotropic) treatment of cell s.u.'s is used for estimating s.u.'s involving l.s. planes.
Refinement. Refinement of F^2^ against ALL reflections. The weighted R-factor wR and goodness of fit S are based on F^2^, conventional R-factors R are based on F, with F set to zero for negative F^2^. The threshold expression of F^2^ > 2σ(F^2^) is used only for calculating R-factors(gt) etc. and is not relevant to the choice of reflections for refinement. R-factors based on F^2^ are statistically about twice as large as those based on F, and R- factors based on ALL data will be even larger.

### Fractional atomic coordinates and isotropic or equivalent isotropic displacement parameters (Å^2^)

**Table:**

	*x*	*y*	*z*	*U*_iso_*/*U*_eq_	
O1A	0.2413 (3)	0.94820 (14)	0.28577 (7)	0.0736 (7)	
N1A	0.3747 (3)	0.88060 (15)	0.21179 (8)	0.0664 (7)	
N2A	0.4546 (3)	0.91235 (16)	0.24990 (8)	0.0675 (7)	
H2AB	0.5495	0.9087	0.2514	0.081*	
N3A	0.2794 (4)	0.9594 (2)	0.38283 (10)	0.1008 (12)	
H3AC	0.2101	0.9666	0.3619	0.121*	
C1A	0.3585 (4)	0.8031 (2)	0.14399 (11)	0.0736 (10)	
C2A	0.4237 (5)	0.7575 (2)	0.11367 (12)	0.0910 (13)	
H2AA	0.3697	0.7423	0.0866	0.109*	
C3A	0.5779 (5)	0.7279 (2)	0.11888 (13)	0.0961 (13)	
H3AA	0.6469	0.7598	0.1018	0.115*	
H3AB	0.5837	0.6788	0.1046	0.115*	
C4A	0.6196 (4)	0.72406 (18)	0.17249 (12)	0.0730 (9)	
H4AA	0.5503	0.6900	0.1884	0.088*	
C5A	0.5956 (4)	0.80113 (18)	0.19480 (12)	0.0781 (10)	
H5AA	0.6133	0.7985	0.2296	0.094*	
H5AB	0.6672	0.8353	0.1810	0.094*	
C6A	0.4416 (4)	0.83090 (18)	0.18604 (10)	0.0650 (9)	
C7A	0.3777 (4)	0.94886 (17)	0.28411 (10)	0.0619 (8)	
C8A	0.4679 (4)	0.9879 (2)	0.32151 (12)	0.0722 (10)	
C9A	0.5983 (4)	1.0215 (2)	0.31003 (14)	0.0842 (11)	
H9AA	0.6308	1.0214	0.2779	0.101*	
C10A	0.6841 (6)	1.0562 (2)	0.34572 (18)	0.1142 (16)	
H10A	0.7715	1.0803	0.3374	0.137*	
C11A	0.6381 (6)	1.0544 (3)	0.39265 (18)	0.122 (2)	
H11A	0.6965	1.0761	0.4166	0.146*	
C12A	0.5076 (6)	1.0213 (3)	0.40553 (15)	0.1101 (17)	
H12A	0.4788	1.0212	0.4380	0.132*	
C13A	0.4167 (5)	0.9875 (2)	0.37067 (12)	0.0828 (12)	
C15A	0.2381 (5)	0.9205 (2)	0.42506 (12)	0.0924 (14)	
C16A	0.3442 (6)	0.8805 (3)	0.45099 (14)	0.1184 (19)	
H16A	0.4438	0.8824	0.4425	0.142*	
C17A	0.2956 (8)	0.8376 (3)	0.49022 (17)	0.130 (2)	
H17A	0.3639	0.8098	0.5078	0.155*	
C18A	0.1500 (9)	0.8358 (3)	0.50314 (17)	0.133 (2)	
H18A	0.1205	0.8068	0.5294	0.160*	
C19A	0.0454 (8)	0.8764 (3)	0.47784 (15)	0.1158 (18)	
C20A	0.0899 (5)	0.9205 (2)	0.43834 (13)	0.0904 (12)	
C21A	0.2037 (5)	0.8302 (2)	0.13614 (13)	0.1015 (14)	
H21A	0.1631	0.8072	0.1076	0.152*	
H21B	0.2045	0.8830	0.1319	0.152*	
H21C	0.1440	0.8177	0.1639	0.152*	
C22A	0.7733 (5)	0.6955 (2)	0.18154 (16)	0.0936 (13)	
C23A	0.7884 (6)	0.6531 (3)	0.2257 (2)	0.180 (3)	
H23A	0.8896	0.6379	0.2296	0.270*	
H23B	0.7261	0.6100	0.2240	0.270*	
H23C	0.7595	0.6830	0.2530	0.270*	
C24A	0.8842 (7)	0.7103 (5)	0.1526 (2)	0.229 (4)	
H24A	0.9899	0.6860	0.1562	0.275*	
H24B	0.8867	0.7160	0.1200	0.275*	
C25A	−0.1157 (7)	0.8735 (3)	0.49329 (17)	0.159 (3)	
H25A	−0.1247	0.8442	0.5223	0.238*	
H25B	−0.1737	0.8516	0.4678	0.238*	
H25C	−0.1507	0.9228	0.4995	0.238*	
C26A	−0.0165 (5)	0.9662 (3)	0.41017 (14)	0.1073 (15)	
H26A	0.0245	1.0146	0.4050	0.161*	
H26B	−0.1076	0.9704	0.4280	0.161*	
H26C	−0.0353	0.9431	0.3793	0.161*	
O1B	0.7412 (3)	0.96096 (13)	0.20763 (7)	0.0724 (6)	
N1B	0.8694 (3)	0.88310 (15)	0.27897 (9)	0.0698 (7)	
N2B	0.9491 (3)	0.91252 (16)	0.24059 (8)	0.0678 (7)	
H2BB	1.0424	0.9041	0.2378	0.081*	
N3B	0.7821 (3)	0.96889 (18)	0.10927 (9)	0.0841 (9)	
H3BC	0.7106	0.9812	0.1283	0.101*	
C1B	0.8460 (4)	0.7998 (2)	0.34366 (11)	0.0713 (9)	
C2B	0.9011 (5)	0.7448 (2)	0.36978 (13)	0.0903 (12)	
H2BA	0.8418	0.7261	0.3946	0.108*	
C3B	1.0496 (5)	0.7098 (2)	0.36318 (14)	0.0930 (13)	
H3BA	1.0375	0.6621	0.3473	0.112*	
H3BB	1.0937	0.7011	0.3949	0.112*	
C4B	1.1538 (4)	0.75777 (19)	0.33295 (12)	0.0740 (9)	
H4BA	1.1827	0.7998	0.3534	0.089*	
C5B	1.0720 (4)	0.78947 (19)	0.28944 (11)	0.0724 (9)	
H5BA	1.1368	0.8239	0.2727	0.087*	
H5BB	1.0493	0.7495	0.2671	0.087*	
C6B	0.9309 (4)	0.82876 (19)	0.30236 (11)	0.0670 (9)	
C7B	0.8768 (4)	0.95481 (18)	0.20755 (10)	0.0640 (8)	
C8B	0.9715 (4)	0.99015 (18)	0.16985 (11)	0.0639 (8)	
C9B	1.1097 (4)	1.01853 (19)	0.18149 (13)	0.0792 (10)	
H9BA	1.1428	1.0162	0.2135	0.095*	
C10B	1.1990 (5)	1.0501 (2)	0.14666 (15)	0.0911 (12)	
H10B	1.2905	1.0701	0.1550	0.109*	
C11B	1.1505 (5)	1.0517 (2)	0.09901 (15)	0.0945 (13)	
H11B	1.2109	1.0718	0.0750	0.113*	
C12B	1.0164 (5)	1.0243 (2)	0.08696 (13)	0.0856 (12)	
H12B	0.9860	1.0259	0.0546	0.103*	
C13B	0.9222 (5)	0.9935 (2)	0.12170 (11)	0.0733 (10)	
C15B	0.7459 (4)	0.9258 (2)	0.06847 (10)	0.0733 (10)	
C16B	0.8528 (5)	0.8825 (2)	0.04525 (11)	0.0933 (14)	
H16B	0.9509	0.8842	0.0554	0.112*	
C17B	0.8119 (6)	0.8375 (3)	0.00722 (13)	0.1074 (16)	
H17B	0.8826	0.8081	−0.0081	0.129*	
C18B	0.6666 (7)	0.8355 (3)	−0.00828 (14)	0.1052 (16)	
H18B	0.6409	0.8048	−0.0341	0.126*	
C19B	0.5590 (6)	0.8780 (2)	0.01349 (12)	0.0891 (12)	
C20B	0.5990 (5)	0.9243 (2)	0.05283 (11)	0.0782 (10)	
C21B	0.6965 (4)	0.8320 (2)	0.35377 (13)	0.0942 (12)	
H21D	0.6567	0.8102	0.3829	0.141*	
H21E	0.6316	0.8218	0.3269	0.141*	
H21F	0.7051	0.8846	0.3580	0.141*	
C22B	1.2935 (5)	0.7160 (2)	0.32092 (16)	0.0924 (12)	
C23B	1.3893 (7)	0.6953 (4)	0.3598 (2)	0.194 (3)	
H23D	1.4841	0.6813	0.3470	0.291*	
H23E	1.3471	0.6542	0.3772	0.291*	
H23F	1.4013	0.7365	0.3816	0.291*	
C24B	1.3230 (7)	0.6901 (4)	0.27603 (19)	0.191 (3)	
H24C	1.2857	0.7072	0.2426	0.229*	
H24D	1.4223	0.6670	0.2761	0.229*	
C25B	0.4013 (6)	0.8752 (3)	−0.00426 (16)	0.125 (2)	
H25D	0.3961	0.8458	−0.0333	0.187*	
H25G	0.3675	0.9246	−0.0111	0.187*	
H25E	0.3396	0.8535	0.0204	0.187*	
C26B	0.4872 (4)	0.9721 (2)	0.07800 (14)	0.0997 (13)	
H26D	0.5257	1.0215	0.0812	0.150*	
H26E	0.4666	0.9522	0.1097	0.150*	
H26F	0.3976	0.9734	0.0592	0.150*	

### Atomic displacement parameters (Å^2^)

**Table:**

	*U*^11^	*U*^22^	*U*^33^	*U*^12^	*U*^13^	*U*^23^
O1A	0.0700 (18)	0.0877 (18)	0.0632 (12)	0.0027 (13)	−0.0034 (11)	−0.0075 (11)
N1A	0.0782 (19)	0.0627 (17)	0.0584 (13)	−0.0030 (15)	−0.0032 (13)	−0.0049 (13)
N2A	0.0708 (19)	0.0670 (19)	0.0649 (14)	−0.0004 (14)	−0.0046 (12)	−0.0146 (13)
N3A	0.098 (3)	0.145 (3)	0.0598 (16)	0.015 (3)	−0.0047 (16)	−0.0036 (19)
C1A	0.092 (3)	0.067 (2)	0.0613 (17)	0.001 (2)	−0.0129 (17)	−0.0092 (16)
C2A	0.118 (4)	0.087 (3)	0.068 (2)	0.006 (3)	−0.025 (2)	−0.0193 (19)
C3A	0.123 (4)	0.084 (3)	0.081 (2)	0.011 (3)	−0.007 (2)	−0.022 (2)
C4A	0.086 (3)	0.0542 (19)	0.0786 (19)	−0.0020 (17)	−0.0021 (18)	−0.0001 (16)
C5A	0.095 (3)	0.059 (2)	0.080 (2)	0.0024 (19)	−0.0089 (19)	−0.0181 (16)
C6A	0.078 (2)	0.059 (2)	0.0576 (16)	−0.0044 (17)	−0.0033 (15)	−0.0015 (15)
C7A	0.070 (2)	0.0554 (19)	0.0604 (16)	0.0051 (16)	−0.0064 (15)	−0.0033 (14)
C8A	0.072 (2)	0.068 (2)	0.077 (2)	0.0162 (18)	−0.0140 (17)	−0.0227 (17)
C9A	0.074 (3)	0.075 (2)	0.104 (3)	0.009 (2)	−0.009 (2)	−0.028 (2)
C10A	0.100 (3)	0.091 (3)	0.152 (4)	0.001 (3)	−0.028 (3)	−0.052 (3)
C11A	0.116 (4)	0.125 (4)	0.124 (4)	0.025 (4)	−0.038 (3)	−0.071 (3)
C12A	0.121 (4)	0.124 (4)	0.085 (3)	0.033 (3)	−0.022 (3)	−0.036 (3)
C13A	0.086 (3)	0.091 (3)	0.072 (2)	0.022 (2)	−0.0162 (19)	−0.0193 (19)
C15A	0.138 (4)	0.083 (3)	0.0559 (18)	0.024 (3)	−0.020 (2)	−0.0173 (18)
C16A	0.171 (5)	0.115 (4)	0.069 (2)	0.042 (4)	−0.015 (3)	−0.022 (2)
C17A	0.199 (7)	0.113 (4)	0.077 (3)	0.034 (5)	−0.020 (4)	−0.012 (3)
C18A	0.228 (8)	0.104 (4)	0.067 (3)	0.003 (5)	−0.018 (4)	0.001 (3)
C19A	0.172 (6)	0.107 (4)	0.069 (2)	−0.025 (4)	−0.004 (3)	−0.021 (3)
C20A	0.115 (4)	0.088 (3)	0.068 (2)	−0.001 (3)	−0.002 (2)	−0.024 (2)
C21A	0.109 (4)	0.109 (3)	0.087 (3)	0.006 (3)	−0.021 (2)	−0.023 (2)
C22A	0.109 (4)	0.067 (3)	0.106 (3)	0.002 (2)	−0.011 (3)	−0.017 (2)
C23A	0.143 (5)	0.121 (5)	0.276 (8)	−0.016 (4)	−0.075 (5)	0.085 (5)
C24A	0.099 (5)	0.421 (13)	0.167 (6)	0.082 (7)	0.018 (4)	0.068 (8)
C25A	0.197 (7)	0.182 (6)	0.098 (3)	−0.065 (6)	0.015 (4)	−0.008 (4)
C26A	0.108 (4)	0.117 (4)	0.097 (3)	0.008 (3)	−0.007 (2)	−0.013 (3)
O1B	0.0701 (18)	0.0785 (16)	0.0686 (12)	−0.0017 (13)	0.0039 (11)	0.0031 (11)
N1B	0.082 (2)	0.0648 (18)	0.0626 (14)	−0.0070 (16)	0.0047 (13)	0.0073 (13)
N2B	0.073 (2)	0.0683 (19)	0.0623 (14)	0.0008 (15)	0.0036 (13)	0.0092 (13)
N3B	0.081 (2)	0.108 (3)	0.0635 (15)	0.0155 (19)	0.0035 (14)	−0.0139 (16)
C1B	0.080 (2)	0.067 (2)	0.0670 (18)	−0.0037 (18)	0.0095 (16)	0.0067 (16)
C2B	0.104 (3)	0.086 (3)	0.081 (2)	−0.004 (2)	0.027 (2)	0.010 (2)
C3B	0.102 (3)	0.089 (3)	0.088 (2)	0.009 (2)	0.022 (2)	0.024 (2)
C4B	0.086 (3)	0.062 (2)	0.0742 (19)	−0.0055 (18)	−0.0005 (17)	0.0027 (16)
C5B	0.077 (2)	0.071 (2)	0.0693 (18)	−0.0075 (18)	0.0107 (16)	0.0059 (16)
C6B	0.080 (2)	0.062 (2)	0.0588 (16)	−0.0084 (18)	0.0014 (16)	0.0013 (15)
C7B	0.076 (3)	0.055 (2)	0.0606 (16)	−0.0002 (17)	0.0009 (15)	−0.0043 (14)
C8B	0.072 (2)	0.0546 (19)	0.0650 (17)	0.0072 (15)	0.0078 (15)	0.0021 (15)
C9B	0.086 (3)	0.066 (2)	0.085 (2)	0.003 (2)	0.008 (2)	0.0115 (18)
C10B	0.077 (3)	0.079 (3)	0.117 (3)	−0.004 (2)	0.016 (2)	0.020 (2)
C11B	0.098 (3)	0.084 (3)	0.101 (3)	0.016 (3)	0.034 (2)	0.030 (2)
C12B	0.098 (3)	0.087 (3)	0.071 (2)	0.018 (2)	0.017 (2)	0.014 (2)
C13B	0.086 (3)	0.072 (2)	0.0616 (17)	0.015 (2)	0.0103 (17)	0.0042 (16)
C15B	0.087 (3)	0.082 (3)	0.0509 (15)	0.012 (2)	0.0037 (16)	0.0110 (16)
C16B	0.118 (4)	0.098 (3)	0.0641 (19)	0.034 (3)	0.005 (2)	0.003 (2)
C17B	0.154 (5)	0.098 (3)	0.071 (2)	0.019 (3)	0.020 (3)	−0.012 (2)
C18B	0.151 (5)	0.100 (3)	0.065 (2)	−0.017 (3)	0.013 (3)	−0.007 (2)
C19B	0.118 (4)	0.087 (3)	0.0628 (19)	−0.009 (3)	−0.001 (2)	0.0129 (19)
C20B	0.101 (3)	0.070 (2)	0.0643 (18)	0.004 (2)	0.0068 (19)	0.0105 (17)
C21B	0.091 (3)	0.098 (3)	0.094 (2)	0.005 (2)	0.031 (2)	0.015 (2)
C22B	0.094 (3)	0.077 (3)	0.106 (3)	0.006 (2)	0.011 (2)	0.017 (2)
C23B	0.138 (6)	0.254 (8)	0.190 (6)	0.068 (6)	−0.049 (5)	−0.025 (6)
C24B	0.199 (6)	0.262 (8)	0.113 (4)	0.137 (6)	0.021 (4)	0.001 (5)
C25B	0.138 (5)	0.130 (4)	0.107 (3)	−0.044 (4)	−0.024 (3)	0.017 (3)
C26B	0.088 (3)	0.101 (3)	0.110 (3)	0.002 (3)	0.004 (2)	0.001 (3)

### Geometric parameters (Å, º)

**Table:**

O1A—C7A	1.232 (4)	O1B—C7B	1.230 (4)
N1A—C6A	1.292 (4)	N1B—C6B	1.297 (4)
N1A—N2A	1.394 (3)	N1B—N2B	1.383 (3)
N2A—C7A	1.342 (4)	N2B—C7B	1.354 (4)
N2A—H2AB	0.8600	N2B—H2BB	0.8600
N3A—C13A	1.381 (5)	N3B—C13B	1.383 (5)
N3A—C15A	1.406 (5)	N3B—C15B	1.403 (4)
N3A—H3AC	0.8600	N3B—H3BC	0.8600
C1A—C2A	1.311 (5)	C1B—C2B	1.323 (5)
C1A—C6A	1.466 (4)	C1B—C6B	1.466 (4)
C1A—C21A	1.496 (5)	C1B—C21B	1.496 (5)
C2A—C3A	1.499 (6)	C2B—C3B	1.494 (5)
C2A—H2AA	0.9300	C2B—H2BA	0.9300
C3A—C4A	1.522 (5)	C3B—C4B	1.525 (5)
C3A—H3AA	0.9700	C3B—H3BA	0.9700
C3A—H3AB	0.9700	C3B—H3BB	0.9700
C4A—C22A	1.501 (5)	C4B—C22B	1.506 (5)
C4A—C5A	1.535 (4)	C4B—C5B	1.517 (4)
C4A—H4AA	0.9800	C4B—H4BA	0.9800
C5A—C6A	1.510 (5)	C5B—C6B	1.501 (5)
C5A—H5AA	0.9700	C5B—H5BA	0.9700
C5A—H5AB	0.9700	C5B—H5BB	0.9700
C7A—C8A	1.489 (4)	C7B—C8B	1.487 (4)
C8A—C9A	1.361 (5)	C8B—C9B	1.387 (5)
C8A—C13A	1.428 (5)	C8B—C13B	1.397 (4)
C9A—C10A	1.398 (5)	C9B—C10B	1.375 (5)
C9A—H9AA	0.9300	C9B—H9BA	0.9300
C10A—C11A	1.355 (6)	C10B—C11B	1.381 (5)
C10A—H10A	0.9300	C10B—H10B	0.9300
C11A—C12A	1.367 (7)	C11B—C12B	1.349 (5)
C11A—H11A	0.9300	C11B—H11B	0.9300
C12A—C13A	1.401 (5)	C12B—C13B	1.394 (5)
C12A—H12A	0.9300	C12B—H12B	0.9300
C15A—C20A	1.387 (6)	C15B—C20B	1.394 (5)
C15A—C16A	1.395 (6)	C15B—C16B	1.396 (5)
C16A—C17A	1.398 (7)	C16B—C17B	1.374 (5)
C16A—H16A	0.9300	C16B—H16B	0.9300
C17A—C18A	1.362 (8)	C17B—C18B	1.380 (7)
C17A—H17A	0.9300	C17B—H17B	0.9300
C18A—C19A	1.384 (7)	C18B—C19B	1.374 (6)
C18A—H18A	0.9300	C18B—H18B	0.9300
C19A—C20A	1.404 (6)	C19B—C20B	1.414 (5)
C19A—C25A	1.516 (7)	C19B—C25B	1.506 (6)
C20A—C26A	1.484 (5)	C20B—C26B	1.497 (5)
C21A—H21A	0.9600	C21B—H21D	0.9600
C21A—H21B	0.9600	C21B—H21E	0.9600
C21A—H21C	0.9600	C21B—H21F	0.9600
C22A—C24A	1.305 (7)	C22B—C24B	1.346 (6)
C22A—C23A	1.443 (6)	C22B—C23B	1.425 (6)
C23A—H23A	0.9600	C23B—H23D	0.9600
C23A—H23B	0.9600	C23B—H23E	0.9600
C23A—H23C	0.9600	C23B—H23F	0.9600
C24A—H24A	1.0556	C24B—H24C	1.0268
C24A—H24B	0.9026	C24B—H24D	0.9881
C25A—H25A	0.9600	C25B—H25D	0.9600
C25A—H25B	0.9600	C25B—H25G	0.9600
C25A—H25C	0.9600	C25B—H25E	0.9600
C26A—H26A	0.9600	C26B—H26D	0.9600
C26A—H26B	0.9600	C26B—H26E	0.9600
C26A—H26C	0.9600	C26B—H26F	0.9600
			
C6A—N1A—N2A	117.1 (3)	C6B—N1B—N2B	116.4 (3)
C7A—N2A—N1A	117.4 (3)	C7B—N2B—N1B	118.5 (3)
C7A—N2A—H2AB	121.3	C7B—N2B—H2BB	120.8
N1A—N2A—H2AB	121.3	N1B—N2B—H2BB	120.8
C13A—N3A—C15A	128.4 (4)	C13B—N3B—C15B	126.0 (3)
C13A—N3A—H3AC	115.8	C13B—N3B—H3BC	117.0
C15A—N3A—H3AC	115.8	C15B—N3B—H3BC	117.0
C2A—C1A—C6A	119.1 (4)	C2B—C1B—C6B	119.4 (4)
C2A—C1A—C21A	122.2 (3)	C2B—C1B—C21B	122.0 (3)
C6A—C1A—C21A	118.7 (3)	C6B—C1B—C21B	118.5 (3)
C1A—C2A—C3A	125.5 (4)	C1B—C2B—C3B	126.1 (3)
C1A—C2A—H2AA	117.2	C1B—C2B—H2BA	116.9
C3A—C2A—H2AA	117.2	C3B—C2B—H2BA	116.9
C2A—C3A—C4A	109.8 (3)	C2B—C3B—C4B	112.3 (3)
C2A—C3A—H3AA	109.7	C2B—C3B—H3BA	109.1
C4A—C3A—H3AA	109.7	C4B—C3B—H3BA	109.1
C2A—C3A—H3AB	109.7	C2B—C3B—H3BB	109.1
C4A—C3A—H3AB	109.7	C4B—C3B—H3BB	109.1
H3AA—C3A—H3AB	108.2	H3BA—C3B—H3BB	107.9
C22A—C4A—C3A	113.8 (3)	C22B—C4B—C5B	115.1 (3)
C22A—C4A—C5A	112.0 (3)	C22B—C4B—C3B	110.6 (3)
C3A—C4A—C5A	108.1 (3)	C5B—C4B—C3B	110.1 (3)
C22A—C4A—H4AA	107.5	C22B—C4B—H4BA	106.9
C3A—C4A—H4AA	107.5	C5B—C4B—H4BA	106.9
C5A—C4A—H4AA	107.5	C3B—C4B—H4BA	106.9
C6A—C5A—C4A	112.9 (3)	C6B—C5B—C4B	113.9 (3)
C6A—C5A—H5AA	109.0	C6B—C5B—H5BA	108.8
C4A—C5A—H5AA	109.0	C4B—C5B—H5BA	108.8
C6A—C5A—H5AB	109.0	C6B—C5B—H5BB	108.8
C4A—C5A—H5AB	109.0	C4B—C5B—H5BB	108.8
H5AA—C5A—H5AB	107.8	H5BA—C5B—H5BB	107.7
N1A—C6A—C1A	115.5 (3)	N1B—C6B—C1B	115.4 (3)
N1A—C6A—C5A	126.1 (3)	N1B—C6B—C5B	127.1 (3)
C1A—C6A—C5A	118.4 (3)	C1B—C6B—C5B	117.3 (3)
O1A—C7A—N2A	122.6 (3)	O1B—C7B—N2B	122.0 (3)
O1A—C7A—C8A	121.7 (3)	O1B—C7B—C8B	122.4 (3)
N2A—C7A—C8A	115.7 (3)	N2B—C7B—C8B	115.6 (3)
C9A—C8A—C13A	120.1 (3)	C9B—C8B—C13B	119.3 (3)
C9A—C8A—C7A	121.6 (3)	C9B—C8B—C7B	121.0 (3)
C13A—C8A—C7A	118.3 (4)	C13B—C8B—C7B	119.7 (3)
C8A—C9A—C10A	121.1 (4)	C10B—C9B—C8B	121.4 (4)
C8A—C9A—H9AA	119.5	C10B—C9B—H9BA	119.3
C10A—C9A—H9AA	119.5	C8B—C9B—H9BA	119.3
C11A—C10A—C9A	119.1 (5)	C9B—C10B—C11B	118.9 (4)
C11A—C10A—H10A	120.4	C9B—C10B—H10B	120.6
C9A—C10A—H10A	120.4	C11B—C10B—H10B	120.6
C10A—C11A—C12A	121.4 (4)	C12B—C11B—C10B	120.6 (4)
C10A—C11A—H11A	119.3	C12B—C11B—H11B	119.7
C12A—C11A—H11A	119.3	C10B—C11B—H11B	119.7
C11A—C12A—C13A	121.2 (4)	C11B—C12B—C13B	121.7 (4)
C11A—C12A—H12A	119.4	C11B—C12B—H12B	119.1
C13A—C12A—H12A	119.4	C13B—C12B—H12B	119.1
N3A—C13A—C12A	121.4 (4)	N3B—C13B—C12B	121.1 (3)
N3A—C13A—C8A	121.5 (3)	N3B—C13B—C8B	120.7 (3)
C12A—C13A—C8A	117.0 (4)	C12B—C13B—C8B	118.1 (4)
C20A—C15A—C16A	121.9 (4)	C20B—C15B—C16B	120.4 (4)
C20A—C15A—N3A	118.2 (4)	C20B—C15B—N3B	118.6 (3)
C16A—C15A—N3A	119.8 (5)	C16B—C15B—N3B	120.9 (3)
C15A—C16A—C17A	117.7 (6)	C17B—C16B—C15B	119.5 (4)
C15A—C16A—H16A	121.2	C17B—C16B—H16B	120.2
C17A—C16A—H16A	121.2	C15B—C16B—H16B	120.2
C18A—C17A—C16A	121.1 (6)	C16B—C17B—C18B	120.3 (5)
C18A—C17A—H17A	119.4	C16B—C17B—H17B	119.8
C16A—C17A—H17A	119.4	C18B—C17B—H17B	119.8
C17A—C18A—C19A	121.0 (6)	C19B—C18B—C17B	121.6 (4)
C17A—C18A—H18A	119.5	C19B—C18B—H18B	119.2
C19A—C18A—H18A	119.5	C17B—C18B—H18B	119.2
C18A—C19A—C20A	119.5 (6)	C18B—C19B—C20B	118.8 (5)
C18A—C19A—C25A	119.8 (5)	C18B—C19B—C25B	120.6 (4)
C20A—C19A—C25A	120.7 (6)	C20B—C19B—C25B	120.6 (5)
C15A—C20A—C19A	118.7 (5)	C15B—C20B—C19B	119.3 (4)
C15A—C20A—C26A	119.2 (4)	C15B—C20B—C26B	119.2 (3)
C19A—C20A—C26A	122.2 (5)	C19B—C20B—C26B	121.5 (4)
C1A—C21A—H21A	109.5	C1B—C21B—H21D	109.5
C1A—C21A—H21B	109.5	C1B—C21B—H21E	109.5
H21A—C21A—H21B	109.5	H21D—C21B—H21E	109.5
C1A—C21A—H21C	109.5	C1B—C21B—H21F	109.5
H21A—C21A—H21C	109.5	H21D—C21B—H21F	109.5
H21B—C21A—H21C	109.5	H21E—C21B—H21F	109.5
C24A—C22A—C23A	123.3 (5)	C24B—C22B—C23B	118.5 (5)
C24A—C22A—C4A	122.6 (5)	C24B—C22B—C4B	122.7 (4)
C23A—C22A—C4A	114.1 (4)	C23B—C22B—C4B	118.3 (4)
C22A—C23A—H23A	109.5	C22B—C23B—H23D	109.5
C22A—C23A—H23B	109.5	C22B—C23B—H23E	109.5
H23A—C23A—H23B	109.5	H23D—C23B—H23E	109.5
C22A—C23A—H23C	109.5	C22B—C23B—H23F	109.5
H23A—C23A—H23C	109.5	H23D—C23B—H23F	109.5
H23B—C23A—H23C	109.5	H23E—C23B—H23F	109.5
C22A—C24A—H24A	123.5	C22B—C24B—H24C	130.6
C22A—C24A—H24B	130.3	C22B—C24B—H24D	108.9
H24A—C24A—H24B	96.7	H24C—C24B—H24D	115.2
C19A—C25A—H25A	109.5	C19B—C25B—H25D	109.5
C19A—C25A—H25B	109.5	C19B—C25B—H25G	109.5
H25A—C25A—H25B	109.5	H25D—C25B—H25G	109.5
C19A—C25A—H25C	109.5	C19B—C25B—H25E	109.5
H25A—C25A—H25C	109.5	H25D—C25B—H25E	109.5
H25B—C25A—H25C	109.5	H25G—C25B—H25E	109.5
C20A—C26A—H26A	109.5	C20B—C26B—H26D	109.5
C20A—C26A—H26B	109.5	C20B—C26B—H26E	109.5
H26A—C26A—H26B	109.5	H26D—C26B—H26E	109.5
C20A—C26A—H26C	109.5	C20B—C26B—H26F	109.5
H26A—C26A—H26C	109.5	H26D—C26B—H26F	109.5
H26B—C26A—H26C	109.5	H26E—C26B—H26F	109.5
			
C6A—N1A—N2A—C7A	−163.2 (3)	C6B—N1B—N2B—C7B	162.9 (3)
C6A—C1A—C2A—C3A	2.4 (6)	C6B—C1B—C2B—C3B	3.4 (6)
C21A—C1A—C2A—C3A	−179.9 (4)	C21B—C1B—C2B—C3B	−178.9 (4)
C1A—C2A—C3A—C4A	28.6 (6)	C1B—C2B—C3B—C4B	17.1 (6)
C2A—C3A—C4A—C22A	−179.5 (3)	C2B—C3B—C4B—C22B	−171.6 (4)
C2A—C3A—C4A—C5A	−54.3 (4)	C2B—C3B—C4B—C5B	−43.4 (5)
C22A—C4A—C5A—C6A	179.9 (3)	C22B—C4B—C5B—C6B	178.2 (3)
C3A—C4A—C5A—C6A	53.7 (4)	C3B—C4B—C5B—C6B	52.5 (4)
N2A—N1A—C6A—C1A	−175.3 (3)	N2B—N1B—C6B—C1B	178.5 (3)
N2A—N1A—C6A—C5A	3.2 (5)	N2B—N1B—C6B—C5B	−6.4 (5)
C2A—C1A—C6A—N1A	174.1 (3)	C2B—C1B—C6B—N1B	−179.5 (3)
C21A—C1A—C6A—N1A	−3.7 (5)	C21B—C1B—C6B—N1B	2.8 (5)
C2A—C1A—C6A—C5A	−4.6 (5)	C2B—C1B—C6B—C5B	4.9 (5)
C21A—C1A—C6A—C5A	177.7 (3)	C21B—C1B—C6B—C5B	−172.8 (3)
C4A—C5A—C6A—N1A	156.9 (3)	C4B—C5B—C6B—N1B	151.3 (3)
C4A—C5A—C6A—C1A	−24.7 (5)	C4B—C5B—C6B—C1B	−33.7 (4)
N1A—N2A—C7A—O1A	8.1 (5)	N1B—N2B—C7B—O1B	−8.2 (5)
N1A—N2A—C7A—C8A	−174.4 (3)	N1B—N2B—C7B—C8B	174.7 (3)
O1A—C7A—C8A—C9A	−146.1 (4)	O1B—C7B—C8B—C9B	144.2 (4)
N2A—C7A—C8A—C9A	36.4 (5)	N2B—C7B—C8B—C9B	−38.7 (5)
O1A—C7A—C8A—C13A	35.8 (5)	O1B—C7B—C8B—C13B	−37.3 (5)
N2A—C7A—C8A—C13A	−141.7 (3)	N2B—C7B—C8B—C13B	139.8 (3)
C13A—C8A—C9A—C10A	−0.2 (6)	C13B—C8B—C9B—C10B	0.3 (6)
C7A—C8A—C9A—C10A	−178.2 (3)	C7B—C8B—C9B—C10B	178.8 (3)
C8A—C9A—C10A—C11A	1.9 (7)	C8B—C9B—C10B—C11B	−1.6 (6)
C9A—C10A—C11A—C12A	−2.0 (8)	C9B—C10B—C11B—C12B	1.5 (6)
C10A—C11A—C12A—C13A	0.4 (8)	C10B—C11B—C12B—C13B	−0.1 (7)
C15A—N3A—C13A—C12A	−39.7 (7)	C15B—N3B—C13B—C12B	45.2 (6)
C15A—N3A—C13A—C8A	144.6 (4)	C15B—N3B—C13B—C8B	−136.8 (4)
C11A—C12A—C13A—N3A	−174.6 (4)	C11B—C12B—C13B—N3B	176.8 (4)
C11A—C12A—C13A—C8A	1.3 (7)	C11B—C12B—C13B—C8B	−1.3 (6)
C9A—C8A—C13A—N3A	174.5 (4)	C9B—C8B—C13B—N3B	−176.9 (3)
C7A—C8A—C13A—N3A	−7.4 (6)	C7B—C8B—C13B—N3B	4.5 (5)
C9A—C8A—C13A—C12A	−1.4 (6)	C9B—C8B—C13B—C12B	1.2 (5)
C7A—C8A—C13A—C12A	176.7 (4)	C7B—C8B—C13B—C12B	−177.4 (3)
C13A—N3A—C15A—C20A	157.1 (4)	C13B—N3B—C15B—C20B	−160.8 (3)
C13A—N3A—C15A—C16A	−25.8 (6)	C13B—N3B—C15B—C16B	22.1 (6)
C20A—C15A—C16A—C17A	2.4 (6)	C20B—C15B—C16B—C17B	−1.0 (6)
N3A—C15A—C16A—C17A	−174.6 (4)	N3B—C15B—C16B—C17B	176.1 (4)
C15A—C16A—C17A—C18A	−1.0 (8)	C15B—C16B—C17B—C18B	0.7 (7)
C16A—C17A—C18A—C19A	0.0 (9)	C16B—C17B—C18B—C19B	−0.1 (8)
C17A—C18A—C19A—C20A	−0.2 (8)	C17B—C18B—C19B—C20B	−0.2 (7)
C17A—C18A—C19A—C25A	−179.7 (6)	C17B—C18B—C19B—C25B	179.7 (4)
C16A—C15A—C20A—C19A	−2.7 (6)	C16B—C15B—C20B—C19B	0.7 (5)
N3A—C15A—C20A—C19A	174.4 (3)	N3B—C15B—C20B—C19B	−176.5 (3)
C16A—C15A—C20A—C26A	177.9 (4)	C16B—C15B—C20B—C26B	−179.1 (3)
N3A—C15A—C20A—C26A	−5.1 (6)	N3B—C15B—C20B—C26B	3.7 (5)
C18A—C19A—C20A—C15A	1.5 (6)	C18B—C19B—C20B—C15B	−0.1 (6)
C25A—C19A—C20A—C15A	−179.0 (4)	C25B—C19B—C20B—C15B	−180.0 (4)
C18A—C19A—C20A—C26A	−179.1 (4)	C18B—C19B—C20B—C26B	179.7 (4)
C25A—C19A—C20A—C26A	0.4 (7)	C25B—C19B—C20B—C26B	−0.2 (6)
C3A—C4A—C22A—C24A	35.6 (7)	C5B—C4B—C22B—C24B	−18.2 (7)
C5A—C4A—C22A—C24A	−87.4 (7)	C3B—C4B—C22B—C24B	107.3 (6)
C3A—C4A—C22A—C23A	−146.6 (4)	C5B—C4B—C22B—C23B	169.7 (5)
C5A—C4A—C22A—C23A	90.4 (5)	C3B—C4B—C22B—C23B	−64.9 (6)

### Hydrogen-bond geometry (Å, º)

**Table:**

*D*—H···*A*	*D*—H	H···*A*	*D*···*A*	*D*—H···*A*
N2*A*—H2*AB*···O1*B*	0.86	2.31	2.969 (4)	134
N3*A*—H3*AC*···O1*A*	0.86	2.14	2.697 (3)	122
N2*B*—H2*BB*···O1*A*^i^	0.86	2.36	2.986 (4)	130
C5*A*—H5*AB*···O1*B*	0.97	2.47	3.189 (4)	130
C9*A*—H9*AA*···O1*B*	0.93	2.43	3.282 (4)	152
C5*B*—H5*BA*···O1*A*^i^	0.97	2.46	3.248 (4)	138
C9*B*—H9*BA*···O1*A*^i^	0.93	2.50	3.352 (4)	153

Symmetry code: (i) *x*+1, *y*, *z*.
